# Lipopolysaccharide preconditioning enhances the efficacy of mesenchymal stem cells transplantation in a rat model of acute myocardial infarction

**DOI:** 10.1186/1423-0127-16-74

**Published:** 2009-08-20

**Authors:** Yongwei Yao, Fumin Zhang, Liansheng Wang, Guohui Zhang, Zhaojun Wang, Jianmei Chen, Xiang Gao

**Affiliations:** 1 Department of Cardiology, First Affiliated Hospital of Nanjing Medical University, 300 Guangzhou Road, Nanjing 210029, PR China; 2 Department of Cardiology, The Affiliated Zhengjiang First People's Hospital of Jiangsu University, 8 Dianli Road, Zhengjiang 212000, PR China; 3 Model Animal Research Center, Nanjing University, 12 Xuefu Road, Pu Kou district, Nanjing 210061, PR China

## Abstract

**Background:**

Mesenchymal stem cells (MSCs)-based regenerative therapy is currently regarded as an alternative approach to salvage the acute myocardial infarcted hearts. However, the efficiency of MSCs transplantation is limited by lower survival rate of engrafted MSCs. In previous study, we found that 1.0 μg/ml Lipopolysaccharide (LPS) could protect MSCs against apoptosis induced by oxidative stress and meanwhile enhance the proliferation of MSCs. Therefore, in the present study, we firstly preconditioned MSCs with 1.0 μg/ml LPS, then transplanted MSCs into ischemic myocardium, and observed the survival and cardiac protective capacity of MSCs in a rat model of acute myocardial infarction. Furthermore, we tried to explore the underlying mechanisms and the role of Toll-like receptor-4 (TLR4) in the signal pathway of LPS-induced cardiac protection.

**Methods and results:**

Acute myocardial infarction model was developed by left anterior descending coronary artery ligation. 60 rats were divided into 4 groups randomly and given an intramyocardial injection of one of the following treatments: 30 μl PBS (control group), 3 × 10^6 ^wild MSCs/30 μl (wMSCs group), 3 × 10^6 ^LPS-preconditioned wild MSCs/30 μl (LPS-wMSCs group), or 3 × 10^6 ^LPS-preconditioned TLR4 gene deleted MSCs/30 μl (LPS-tMSCs group). After 3 weeks, LPS-preconditioned wild MSCs transplantation ameliorated cardiac function and reduced fibrosis of infarcted myocardium. Vascular density was markedly increased in LPS-wMSCs group compared with other three groups. Survival rate of engrafted MSCs was elevated and apoptosis of myocardium was reduced in infarcted heart. Expression of vascular endothelial growth factor (VEGF) and phospho-Akt was increased in the infarcted myocardium after transplantation of LPS-preconditioned MSCs.

**Conclusion:**

LPS preconditioning enhanced survival of engrafted MSCs, stimulated expression of VEGF and activated PI3K/Akt pathway. LPS preconditioning before MSCs transplantation resulted in superior therapeutic neovascularization and recovery of cardiac function. LPS preconditioning provided a novel strategy in maximizing biologic and functional properties of MSCs.

## Background

Despite the clinical success of reperfusion therapy, a large proportion of patients develop myocardial necrosis, ventricular remodelling and resultant congestive heart failure after acute myocardial infarction (AMI) [1]. Mesenchymal stem cells (MSCs)-based regenerative therapy is currently regarded as an alternative approach to salvage the MI hearts [2-4]. However, a major challenge to MSCs therapy is that transplanted cells undergo apoptosis, as they are exposed to an extremely harsh microenvironment in the infarcted heart. The efficiency of MSCs transplantation is limited by lower survival rate of MSCs [5,6]. Therefore, protection of MSCs against apoptosis is critical for successful cellular therapy. Novel strategies are being developed to maximize biologic and functional properties of MSCs, such as the preparation of cells in special bioscaffolds [7], preconditioning of cells in culture [8,9], and genetic transfection [10,11].

Toll-like receptors (TLRs) are a class of molecules which play an important role in the innate immune system for the recognition of pathogen-associated molecular patterns by immune cells, initiating a primary response toward invading pathogen and recruitment of the adaptive immune response [12-14]. Recent research found that MSCs express TLR molecules 1 to 8. Pam3Cys, a prototypic ligand for TLR2, induced proliferation of MSCs and regulated their differentiation [15]. Lipopolysaccharide (LPS) is the antigenic component of the gram-negative bacterial cell wall and is known as the ligand of Toll-like receptor-4 (TLR4). In our previous study, we found that 1.0 μg/ml LPS could protect MSCs against apoptosis induced by oxidative stress, and enhance the proliferation of MSCs [16]. However, whether LPS could protect MSCs against apoptosis and increase the survival rate of engrafted MSCs in the infarcted myocardium need to be further investigated.

In the present study, we preconditioned MSCs with 1.0 μg/ml LPS, then transplanted them into ischemic myocardium, and observed the survival and cardiac protective capacity of MSCs in a rat acute myocardial infarction model. Furthermore, we tried to explore potential mechanisms and the role of TLR4 in the signal pathway of LPS-induced cardiac protection.

## Methods

### Animal preparation

Adult male C57BL/10J mice (wild type, 6-8 weeks, 25-30 g) and C57BL/10ScNJ mice (TLR4 gene deleted type, 6-8 weeks, 25-30 g) were provided by the Model Animal Research Centre of Nanjing University (Nanjing, China). Adult female Wistar rats (210-250 g) were purchased from Slac company (Shanghai, China). The procedure was performed in accordance with the "Guide for the Care and Use of Laboratory Animals" (NIH Publication No. 85-23, National Academy Press, Washington, DC, revised 1996). The study protocol was approved by the Animal Care and Use Committee of Nanjing Medical University.

### Isolation, expansion and labeling of MSCs

Wild MSCs (wMSCs) were harvested from male C57BL/10J mice, and TLR4 gene deleted MSCs (tMSCs) were isolated from male C57BL/10ScNJ mice, respectively. Isolation of MSCs from mice was performed as previously described with minor modifications [17]. Briefly, mice were sacrificed humanely and femurs were aseptically harvested. Whole marrow cells were obtained by flushing the bone marrow cavity with low glucose Dulbecco's Modified Eagle's Medium (L-DMEM, Hyclone, USA). Cells were centrifuged at 1000 × *g *for 5 minutes and the supernatant was removed. The cell pellet was then re-suspended with L-DMEM supplemented with 10% fetal bovine serum (FBS, Hyclone, USA), 100 U/ml penicillin (Invitrogen), 100 U/ml streptomycin (Invitrogen), and incubated at 37°C in a 5% CO_2 _atmosphere. After 24 hours, non-adherent cells in suspension were discarded and culture media was replaced every three or four days thereafter. When MSCs reached 80%-90% of confluence, they were trypsinized by the addition of 0.25% trypsin-EDTA (Sigma-Aldrich, USA), and then re-plated in culture flasks at a density of 5 × 10^4^/cm^2^. Cells between passages 4 and 5 were utilized for experiment. Cells were confirmed to be MSCs by cell surface markers with flow cytometry analysis: CD14^-^; CD29^+^; CD34^-^; CD44^+^; CD45^-^, and CD105^+^.

To label MSCs with 1,1'-dioctadecyl-3,3,3'3'-testramethylindo-carbocyanine perchlorate (DiI), 2 μg/ml DiI was added to MSCs suspension and incubated at 37°C for 5 minutes, then at 4°C for 15 minutes with occasional mixing. MSCs labeled with DiI were washed 3 times with PBS. All cells were kept frozen until use.

### VEGF assessment by Enzyme-link immunosorbent assay (ELISA)

wMSCs and tMSCs were plated in 24 well plates respectively at a concentration of 1 × 10^5 ^cells/well/ml and incubated with serum-free L-DMEM. After 24 hours, wMSCs and tMSCs were stressed with different concentrations of LPS (0 μg/ml, 0.01 μg/ml, 0.1 μg/ml, 1.0 μg/ml, 10 μg/ml) respectively. Cells were subsequently exposed to 5 nmol/L pyrrolidine dithiocarbamate (PDTC) or not, which was an inhibitor of NF-κB. After 48 hours of incubation, supernatants were harvested. Concentration of vascular endothelial growth factor (VEGF) in supernatants was determined by VEGF ELISA kits (R&D Systems) according to the instruction of the manufacturer. Experiments were repeated three times for verification of results.

### Myocardial infarction model and MSCs transplantation

Female Wistar rats were anesthetized with ketamine (100 mg/kg) by intraperitoneal injection and mechanically ventilated. Under a sterile condition, the heart was exposed through a left thoracotomy, and the left anterior descending coronary artery was ligated at just below the atrioventricular border. Successful ligation was identified by cyanosis of the left ventricular anterior wall and ST-T elevation on electrocardiogram. The rats were allowed to recover under care. Before transplantation, LPS-preconditioned nMSCs and tMSCs were exposed to 1.0 μg/ml LPS for 48 hours and collected. 80 rats were used in our experiment. 12 rats died after AMI, and 8 rats died within 7 days after AMI. One week after AMI, remaining 60 rats were divided into 4 groups randomly and given an intramyocardial injection with one of the following treatments: 30 μl PBS (control group, n = 15), 3 × 10^6 ^wMSCs/30 μl (wMSCs group, n = 15), 3 × 10^6 ^LPS-preconditioned wMSCs/30 μl (LPS-wMSCs group, n = 15), or 3 × 10^6 ^LPS-preconditioned tMSCs/30 μl (LPS-tMSCs group, n = 15). 3 × 10^6 ^MSCs were injected into two sites of the myocardium with a bent 26-gauge needle, one within the infarct area and the other in the myocardium bordering the ischemic area. At the end of experiment, the chest was closed, and animals were weaned from the ventilator and allowed to recover. Three weeks after cells transplantation, the rats were sacrificed and hearts from different groups were harvested for the following experiments.

### Functional assessment by echocardiography

Three weeks after cells transplantation, the rats were undergone cardiac function assessment by transthoracic echocardiography with 12-MHz phased-array transducer (Hewlett Packard). The heart was imaged in the cross-sectional mode in parasternal long- and short-axis views of the left ventricle (LV). Average LV end-diastolic diameter (LVDd), LV end-systolic diameter (LVDs), LV ejection Fraction (LVEF) and LV fractional shortening (FS) were measured from three consecutive cardiac cycles. All measurements were done by two experienced echocardiographer who were blinded to treatment assignment.

### Fibrosis assessment by Masson's trichrome

To detect fibrosis in cardiac muscle, the LV myocardium (n = 5, each) was fixed in 4% paraformaldehyde, cut transversely, embedded in paraffin, and stained with Masson's trichrome. Ten anterolateral sections from each heart were evaluated in their entirety and quantified. Fibrosis was determined by measuring the collagen area as a proportion of the total area under observation. These morphometric studies were performed by two examiners who were blinded to treatment assignment.

### Evaluation of survival of engrafted MSCs

Hearts were harvested, soaked in 30% sucrose in PBS overnight at 4°C and then embedded in Tissue-Tak OCT (Sakura). Serial 20 μm thick sections were cut at -20°C and observed under fluorescence microscope (Leica Microsystems, Wetzlar, Germany). Engrafted MSCs were identified by the presence of DiI-labeled cells.

Because the engrafted cells were harvested from male mice, the survival rate of the transplanted MSCs could be examined by the quantitation of *sry *(sex-determining region of Y-chromosome) gene in the female rat heart, using real-time fluorescence TaqMan PCR 3 weeks after cells transplantation. Briefly, after the animals were killed, hearts from different groups were removed and DNA was isolated using Genomic DNA Isolation Kit (QIAGEN). Specific primers were designed based on the GeneBank sequences of the sex-determining region of the Y chromosome5 of the rat (sense 5'-GAGGCACAAGTTGGCTCAACA-3'; antisense 5'-CTCCTGCAAAAAG-GGCCTTT-3') [18]. Quantitative PCR was performed and analyzed as described previously [19].

### TUNEL assay

Three weeks after cells transplantation, apoptotic cardiomyocytes in the infarcted heart were evaluated by TUNEL assay using an in situ Cell Death Detection Kit (Roche, Germany) according to the manufacturer's instructions. Apoptotic cells were identified by brown colour in their nuclei. Tissue sections were examined microscopically and the percentage of apoptotic cells per total cells was determined in eight randomly chosen fields.

### Immunohistochemical assay

Rats from each group were sacrificed 3 weeks after cells transplantation. Heart muscle samples were fixed in 4% paraformaldehyde, embedded in paraffin, and sectioned at 5 μm intervals. Endothelial cells were identified by the expression of von Willebrand Factor (vWF). Immunohistology was performed with specific primary antibody against vWF (1:100, abcom). Five fields on the slide presenting infarcted area from each heart were randomly chosen for counting stained blood vessels under microscope (×100, Olympus CX-21). The blood vessel density was expressed as vessel number/field.

### Western blot analysis of VEGF, Akt and phospho-Akt

Cytoplasmic protein was prepared from heart tissue and immunoblot was performed as described previously [20]. Protein was analyzed using 10% sodium dodecyl sulphate-polyacrylamide gel electrophoresis (SDS-PAGE) and transferred to nitrocellulose membranes (Bio-Rad). Membranes were then incubated with primary antibodies including VEGF (1:1000, Cell Signaling), Akt (1:1000, Cell Signaling), phosphorylated Akt (1:1000, Cell Signaling) and β-actin (1:5000, Sigma) at 4°C overnight respectively. The membranes were then incubated with peroxidase labeled secondary antibody (1:1000, Santa Cruz, USA) at 37°C for 2 hours. Signals were detected by enhanced chemiluminescence (Amersham, USA). Densitometric analysis for the blots was performed with NIH image software. Experiments were repeated 6 times for verification of results.

### Statistical analysis

Data are expressed as mean ± SEM and analysed with SPSS11.0 statistical software. Differences between groups were analyzed by Student *t *test or Fisher's exact test, where appropriate. Statistical significance was set at < 0.05 level.

## Results

### LPS promoted VEGF secretion of MSCs in vitro

**Figure 1:**
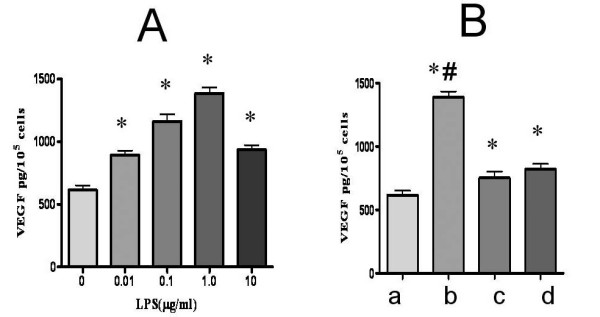
**VEGF concentration in the culture supernant of MSCs**. ELISA analysis of supernatant after stimulation of LPS is shown. Wild MSCs were exposed to increasing doses of LPS for 48 hours. VEGF concentration increased after LPS treatment and VEGF concentration was the highest after incubation with 1.0 μg/ml LPS. B. In the presence of NF-κB inhibitor-PDTC, LPS stimulation caused a subsequent decrease in VEGF expression compared with that without NF-κB inhibition. LPS stimulation on tMSCs caused an obvious decrease in VEGF expression compared with that of wild MSCs. (a. VEGF concentration of wMSCs without stimulation of LPS; b. VEGF concentration of wMSCs with 1.0 μg/ml LPS stimulation; c. VEGF concentration of wMSCs with stimulation of 1.0 μg/ml LPS and 5 nmol/L PDTC; d. VEGF concentration of TLR4 gene deleted MSCs with stimulation of 1.0 μg/ml LPS) Data are mean ± SEM. *P < 0.05 vs. control group, ^#^*P *< 0.05 vs. NF-κB inhibition and TLR4 deletion groups.

VEGF concentration was higher in the groups following LPS treatment than that without it (concentration of LPS is 0, 0.01, 0.1, 1.0, 10.0 μg/ml and corresponding concentration of VEGF is 615.5 ± 36.7, 892.7 ± 35.3, 1163 ± 55.8, 1387 ± 48.1, 938.3 ± 35.2 pg/10^5^cells). VEGF concentration was the highest after incubation with 1.0 μg/ml LPS for 48 hours (Fig 1A). In the presence of NF-κB inhibitor-PDTC, LPS stimulation induced increase in VEGF expression was reduced (718.3 ± 32.7 vs.1387 ± 48.1 pg/10^5^cells, respectively, *P *< 0.01). LPS induced stimulation of VEGF expression in tMSCs is less than that of wild MSCs (760 ± 38.2 vs.1387 ± 48.1 pg/10^5^cells, respectively, *P *< 0.01, Fig 1B).

### LPS-preconditioned MSCs improved cardiac function

Three weeks after transplantation, LPS-wMSCs group had greater LVEF than wMSCs group and LPS-tMSCs group (*P *< 0.01). These three groups had significantly greater LVEF than control group (*P *< 0.01), whereas without difference between wMSCs group and LPS-tMSCs group. Other echocardiographic parameters, including FS, LVDd and LVDs, showed similar difference among groups (table 1). The results suggested that an improved cardiac function was obtained by injection of LPS-pretreated wMSCs.

**Table 1:** Cardiac function 3 weeks after surgery.

	LVDd(cm)	LVSd(cm)	LVEF(%)	FS(%)
control group	0.78 ± 0.02	0.68 ± 0.03	30.66 ± 1.56	11.21 ± 0.45
wMSCs group	0.70 ± 0.02^a^	0.54 ± 0.03^a^	51.18 ± 0.97^a^	21.13 ± 0.55^a^
LPS-wMSCs group	0.68 ± 0.02^a, b, c^	0.46 ± 0.02^a, b, c^	65.43 ± 1.25^a, b, c^	25.88 ± 0.54^a, b, c^
LPS-tMSCs group	0.71 ± 0.02^a^	0.57 ± 0.02^a^	50.38 ± 1.29^a^	20.91 ± 0.71^a^

Note: ^a^*P *< 0.01 vs. control group; ^b^*P *< 0.01 vs. wMSCs group; ^c^*P *< 0.01 vs. LPS-tMSCs group.

### LPS-preconditioned MSCs reduced the fibrosis of infarcted myocardium

**Figure 2:**
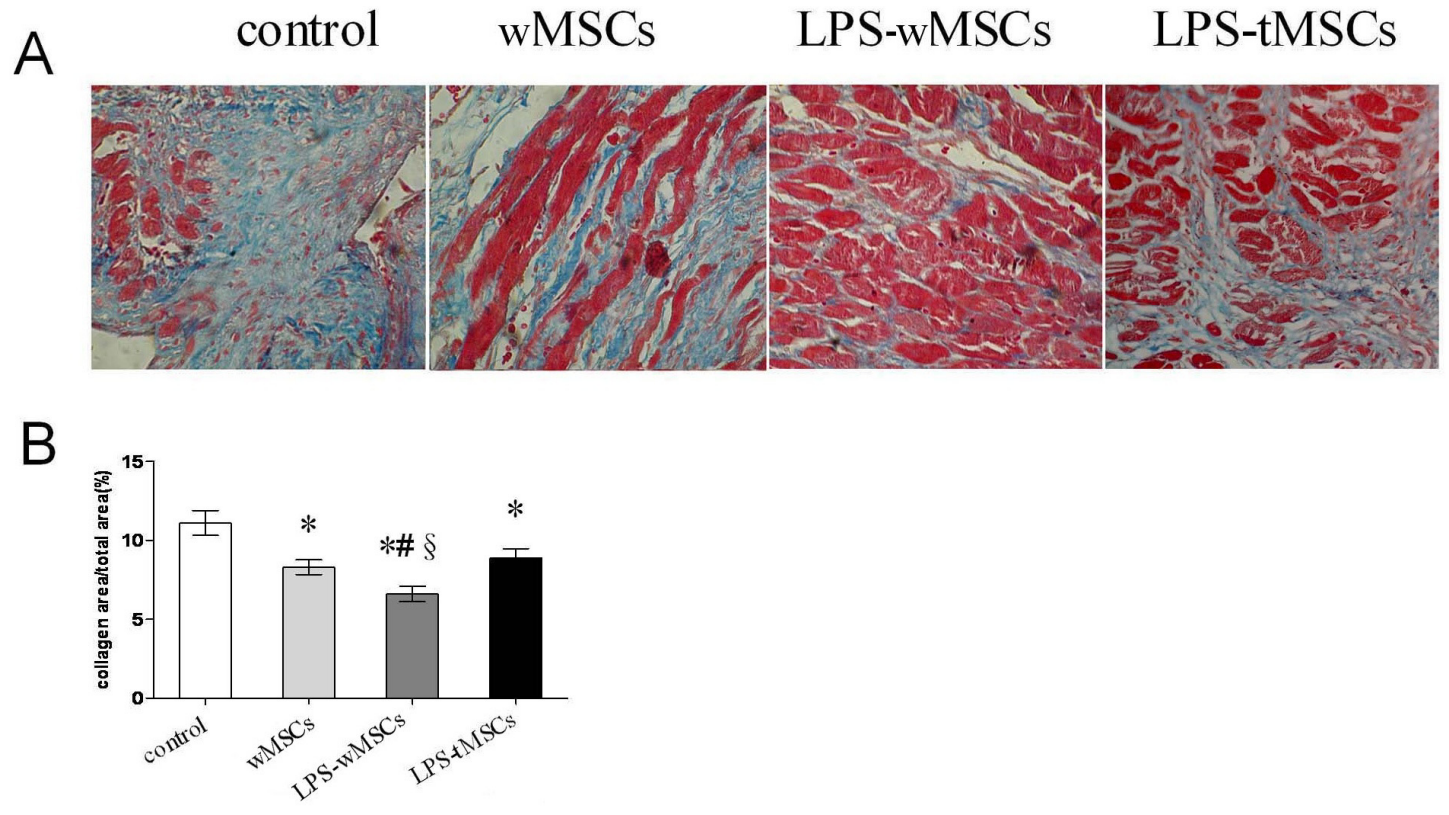
**LPS-preconditioned MSCs reduced the fibrosis of infarcted myocardium**. Representative samples of myocardial fibrosis stained with Masson's trichrome (× 400). B. Quantitative analysis of myocardial fibrosis. Data are mean ± SEM. * *P *< 0.05 versus control group; ^#^**P **< 0.05 versus wMSCs group; ^§^*P *< 0.05 versus LPS-tMSCs group.

Masson's trichrome staining demonstrated modest myocardial fibrosis in the control group (11.1 ± 0.8%). wMSCs and LPS-preconditioned tMSCs significantly reduced fibrosis (8.3 ± 0.5% and 8.9 ± 0.6%, *P *< 0.05), whereas LPS-preconditioned wMSCs reduced fibrosis further (6.6 ± 0.5%, *P *< 0.05). There was no statistical significance between wMSCs group and LPS-tMSCs group (Fig 2).

### Survival of transplanted cells

**Figure 3:**
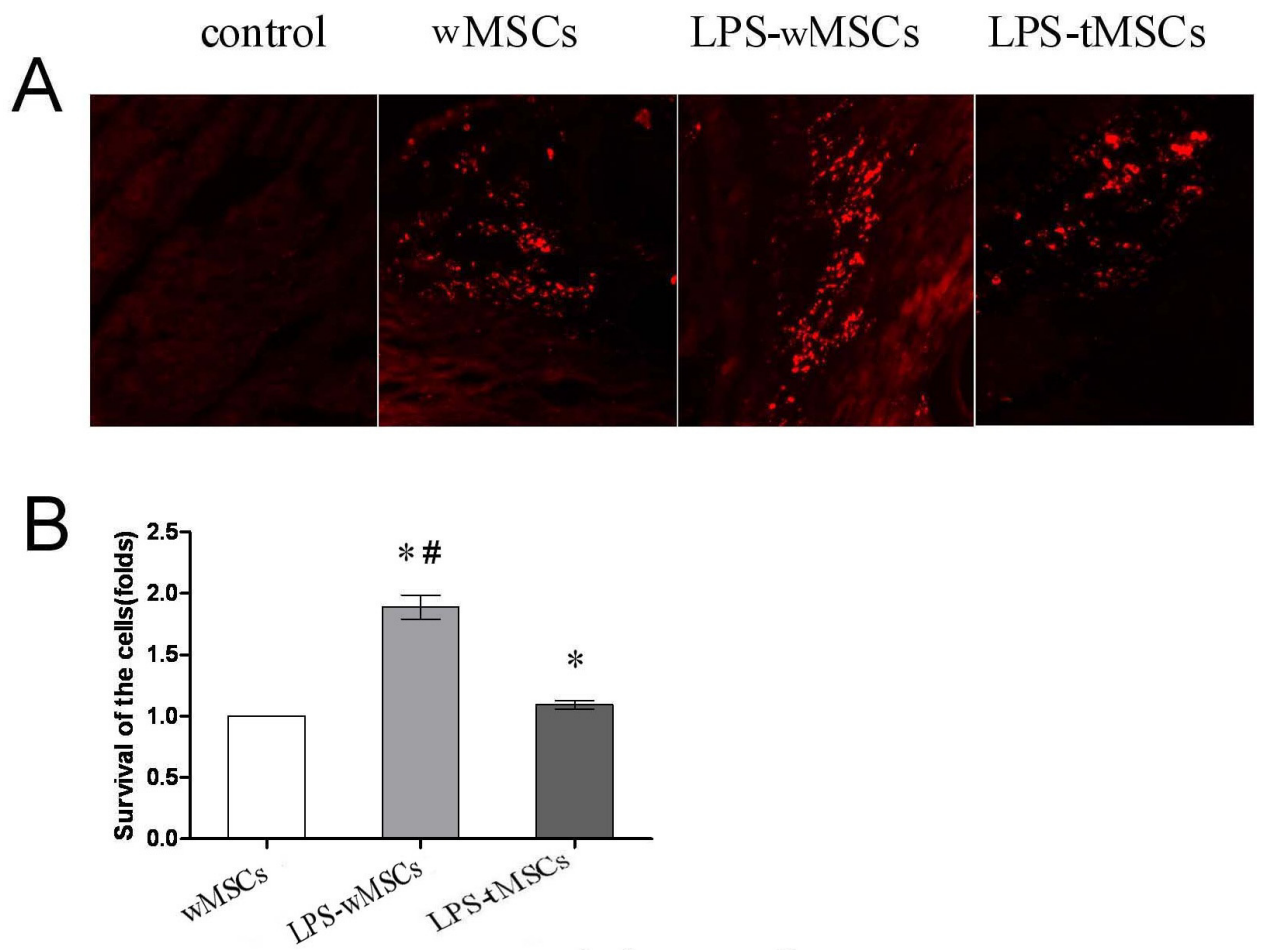
**LPS preconditioning enhanced the survival of engrafted MSCs**. A. Engrafted MSCs were detected by the presence of DiI-labeled cells (red) and cells were not found in control group(× 200). B. Real-time PCR for *sry *gene in the female heart tissue samples at 3 weeks after sex-mismatched cell transplantation. The fold change in *sry *gene expression in different groups was calculated. The result showed significantly higher survival rate of the engrafted cells in the LPS-wMSCs group compared with that in wMSCs group and LPS-tMSCs group. Data are mean ± SEM. *P < 0.01 versus wMSCs group; ^#^*P *< 0.01 versus LPS-tMSCs group.

Many engrafted MSCs were detected along the track of needle injection by the presence of DiI-labeled cells. After 3 weeks, a decrease in the absolute number of engrafted cells was observed in the three MSCs transplanted groups, and transplanted cells were not found in control group (Fig 3A). However, real-time PCR for *sry *gene showed that the survival rate of donor cells in LPS-wMSCs group was significantly higher (1.89 ± 0.10-folds) compared with that in wMSCs group (1 fold, *P *< 0.01). The survival of the donor cells in LPS-tMSCs group was higher (1.09 ± 0.04-folds) than that in wMSCs group, but did not show significant statistical difference (Fig 3B). These results indicate that LPS preconditioning can enhance the survival of engrafted MSCs in the infarcted heart.

### LPS-preconditioned MSCs transplantation reduced the apoptosis of myocardium

**Figure 4:**
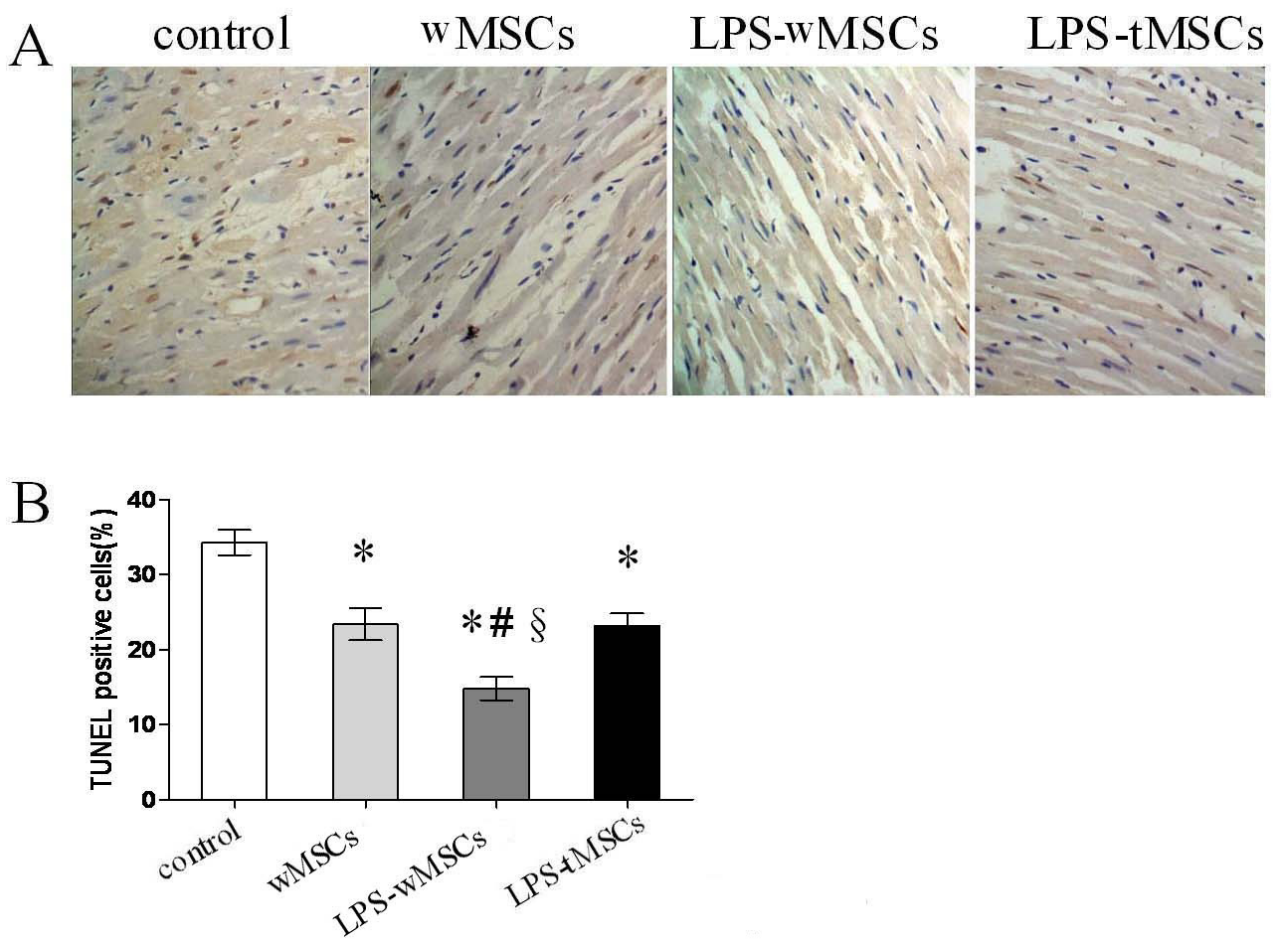
**LPS-preconditioned MSCs transplantation reduced the apoptosis of myocardium**. A. Representative photo-micrographs of TUNEL-positive nuclei staining (brown) in ischemic cardiac muscle cells (× 400). B. The percentage of apoptotic cells was the lowest in LPS-wMSCs group among the four groups. Data are mean ± SEM. *P < 0.01 versus control group; ^#^*P *< 0.01 versus wMSCs group; ^§^*P *< 0.01 versus LPS-tMSCs group.

TUNEL-positive nuclei staining was detectable in infarcted heart muscle cells in both control and experimental groups (Fig 4A). The percentage of TUNEL-positive cells markedly decreased in LPS-wMSCs group (14.8 ± 1.5%), wMSCs group (23.4 ± 2.1%) and LPS-tMSCs group (23.2 ± 1.6%), compared with that in control group (34.3 ± 1.7%, *P *< 0.01). The percentage of apoptotic cells was the lowest in LPS-wMSCs group among the four groups (*P *< 0.01) (Fig 4B).

### LPS-preconditioned MSCs transplantation increased vascular density

**Figure 5:**
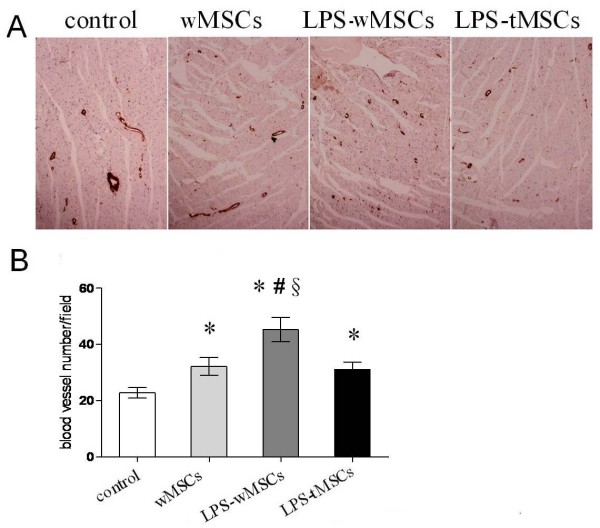
**LPS-preconditioned MSCs transplantation increased vascular density**. A. Endothelial cells were stained with antibody against vWF, and blood vessel numbers were counted (× 100). B. Five fields were randomly selected from each sample (n = 8), and blood vessel density is shown as the vessel number/field. Data are mean ± SEM. *P < 0.01 vs. control group; ^#^*P *< 0.01 vs. wMSCs group; ^§^*P *< 0.01 vs. LPS-tMSCs group.

Immunostaining of the infarcted myocardium for vWF expression showed an augmentation of neovascularization in the cells-transplanted groups (Fig 5A). 3 weeks after cells or PBS injection, blood vessel density per fields in the infarct or peri-infarct zones was significantly greater in LPS-wMSCs group (45.3 ± 4.3, *P *< 0.01) than that in wMSCs group (32.2 ± 3.1) and LPS-tMSCs group(31.1 ± 2.7). Control group (22.8 ± 1.9, *P *< 0.01) had the lowest vessel density among the 4 groups (Fig 5B).

### LPS-preconditioned MSCs transplantation increased expression of VEGF and phosphorylated Akt in vivo

**Figure 6:**
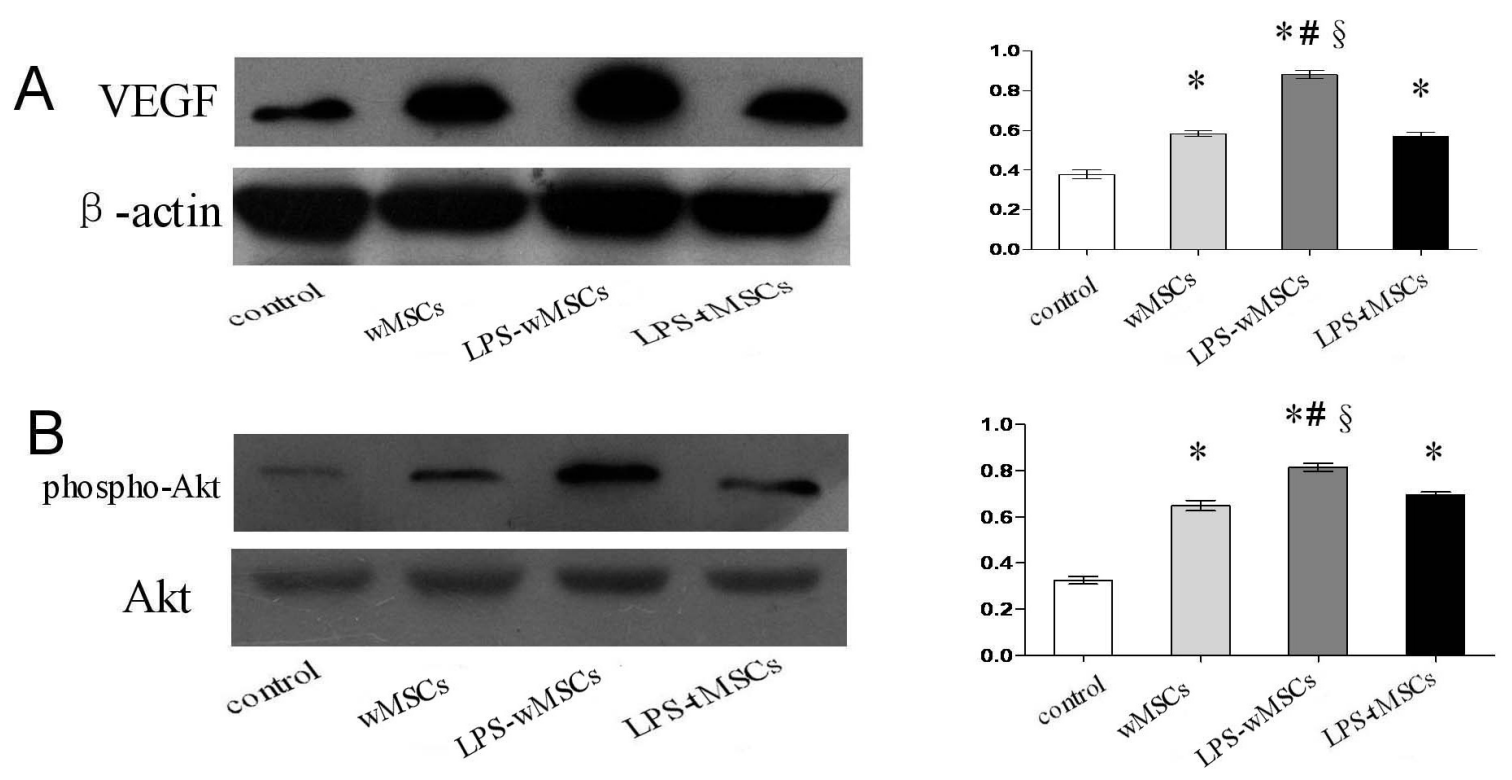
**LPS-preconditioned MSCs transplantation increased expression of VEGF and phosphorylated Akt protein *in vivo***. Representative Western blots for expression of VEGF. Corresponding β-actin blots are shown as a control for sample loading. Bar graph on right shows quantitation. The protein levels for each sample were determined as a ratio to their corresponding β-actin levels. B. Representative Western blots for expression of phospho-Akt, Akt. Total Akt served as a loading control. Bar graph on right shows quantitation. The protein levels of phospho-Akt for each sample were determined as a ratio to their corresponding Akt levels. Data are mean ± SEM. *P < 0.05 versus control group; ^#^*P *< 0.05 versus wMSCs group; ^§^*P *< 0.05 versus LPS-tMSCs group.

To determine if LPS preconditioning improves VEGF secretion *in vivo*, protein was collected from infarcted myocardium. Western blot analysis showed that levels of VEGF were increased in cells transplanted groups compared with that in control group (*P *< 0.05). LPS-wMSCs group had the highest VEGF secretion among the four groups (Fig 6A). To investigate whether LPS preconditioning could induce the activation of PI3K/Akt signalling in the myocardium, we examined the level of phospho-Akt in the myocardium. As shown in Fig 6B, significant increase in the ratio of phospho-Akt/Akt was observed in the cells-transplanted groups compared with that in control group (*P *< 0.05). The ratio of phospho-Akt/Akt was the highest in the LPS-wMSCs group among the four groups (*P *< 0.05). Thus, LPS-pretreated MSCs transplantation increased expression of VEGF and phospho-Akt in infarcted myocardium.

## Discussion

The main findings of this study are 1) LPS preconditioning can improve the survival of MSCs, promote expression of VEGF and phospho-Akt in infarcted myocardium; 2) LPS-preconditioned MSCs transplantation can enhance cardiac function, reduce apoptosis of myocardium, inhibit fibrosis and elevate vascular density after MI; 3) TLR4 plays an important role in the signal pathway of LPS-induced cardiac protection.

Although few reports have quantified stem cell survival after transplantation, donor stem cell survival may be as low as 1%-5% 48 hours after transplantation [2,6], possibly due to a hostile, nutrient-deficient, inflammatory environment within damaged myocardium. This low survival may limit the sustained reparative capacity of stem cells *in vivo*. Enhanced survival of transplanted stem cells may increase their cytoprotective efficacy. Our previous study had suggested that 1.0 μg/ml LPS could protect MSCs from oxidative stress-induced apoptosis and improve the survival of MSCs via TLR4 and PI3K/Akt pathway. After pretreated by 1.0 μg/ml LPS, MSCs strengthened the power against oxidative stress and inflammatory environment in infarcted area. In present study, we also found that survival ratio of transplanted MSCs was elevated in infarcted myocardium after LPS preconditioning by detecting the *sry *gene in the donor heart.

Recent studies have indicated that paracrine mechanism, carried out by the cells through the release of soluble cellular factors, might be a critical mechanism of tissue repair and functional improvement after MSCs transplantation [21]. VEGF is a crucial paracrine factor in MSCs-mediated cardioprotection. In addition to its potent angiogenic function, VEGF can stimulate proliferation, delay senescence, suppress apoptosis and promote survival of various cells [22,23]. It has reported that MSCs exposed to LPS could increase expression of VEGF [24,25]. In *in vitro *study, we demonstrated that a significant level of VEGF was detected in the culture medium of LPS-treated MSCs. In *in vivo *study, Western bolt analysis also implied that LPS-preconditioning MSCs transplantation released more VEGF in infarcted myocardium than that without LPS preconditioning. As a result of VEGF secretion, the vascular density was increased in LPS-preconditioning group than that without LPS-preconditioning. Furthermore, the protected cardiac function and the reduced apoptosis of myocardium in infarcted heart were also related with expression of VEGF. Thus, we demonstrated that LPS-preconditioning promoted the release of VEGF, which in turn improved the effect of MSCs transplantation in the infarcted area.

Phosphoinositide 3-kinase (PI3K) and its downstream target serine/threonine kinase Akt are now recognized as one of the most critical pathways in regulating cellular activation, inflammatory responses and apoptosis [26,27]. Activation of PI3K/Akt-dependent signalling has been shown to prevent cardiac myocyte apoptosis and protect the myocardium from AMI [10,28]. Akt is activated by site-specific phosphorylation. In our study, we found that the level of phosphor-Akt was elevated and the apoptosis of myocardium was reduced in LPS-wMSCs group compared with that in wMSCs group. So we demonstrated that LPS-preconditioned MSCs resulted in significantly increased levels of phospho-Akt in the myocardium which positively correlated with cardioprotection. Our results indicated that effect of LPS-conditioned MSCs transplantation was mediated partially through a PI3K/Akt-dependent mechanism.

TLRs are a conservative family of receptors that recognize pathogen-associated molecular patterns and promote the activation of immune cells [29,30]. Recent studies have shown that the heart expresses TLR4 mRNA and protein [31], and TLR4 plays a role in myocardial dysfunction during bacterial sepsis [32,33]. However, a low dose of LPS (0.2 mg/kg) preconditioning induced cardioprotection in a rodent model of ischaemia/reperfusion injury [34]. Preconditioning, injury-induced protection from subsequent injury, has been proven to be a powerful form of cardioprotection. Jiang et al. [35] preconditioned MSCs with hypoxia, transplanted the stem cells into ischemic myocardium, and observed enhanced survival and cytoprotective capacity. Guo et al. [36] preconditioned MSCs with IGF-1 and found enhancement of the number of MSCs attracted into the infarcted heart. In the present study, we found LPS-preconditioning could enhance survival of MSCs, stimulate expression of VEGF and activate PI3K/Akt pathway. Conversely, TLR4-deleted MSCs (tMSCs) released less VEGF and exerted less functions in reducing apoptosis of myocardium than LPS-preconditioning wMSCs. TLR-mediated signalling activates NF-κB, which plays a critical role in regulating expression of genes, such as VEGF. In our *in vitro *study, we found that NF-κB inhibition prevented secretion of VEGF under the LPS condition. So we concluded that TLR4/NF-κB signalling pathway played an important role in cardioprotection performed by LPS preconditioning. LPS preconditioning could promote expression of other cytokines, such as fibroblast growth factor 2 (FGF2), insulin-like growth factor 1 (IGF-1), and hepatocyte growth factor (HGF) [25]. The exact effects of these cytokines in infarcted heart need a further investigation.

In conclusion, this study demonstrates that LPS preconditioning can enhance survival of MSCs, stimulate expression of VEGF, and activate PI3K/Akt pathway. LPS preconditioning before MSCs transplantation leads to superior therapeutic neovascularization and recovery of cardiac function. Therefore, LPS preconditioning provides a novel strategy in maximizing biologic and functional properties of MSCs.

## Competing interests

The authors declare that they have no competing interests.

## Authors' contributions

YY carried out the main experiment and drafted the manuscript. FZ conceived of the study, designed the experiment and helped to drafted the manuscript. LW performed immunohistochemical assay. GZ finished statistical analysis. ZW participated in Western blot analysis. JC carried out culture of MSCs and helped to finish animal model. XG provided and cultured TLR4 deleted MSCs and helped to perform the real-time PCR. All authors read and approved the final manuscript.
